# Therapeutic hypercapnia for prevention of secondary ischemia after severe subarachnoid hemorrhage: physiological responses to continuous hypercapnia

**DOI:** 10.1038/s41598-021-91007-7

**Published:** 2021-06-03

**Authors:** Christian Stetter, Franziska Weidner, Nadine Lilla, Judith Weiland, Ekkehard Kunze, Ralf-Ingo Ernestus, Ralf Michael Muellenbach, Thomas Westermaier

**Affiliations:** 1 Department of Neurosurgery, University Hospital Wuerzburg, Josef-Schneider-Strasse 11, 97080 Wuerzburg, Germany; 2 Department of Anesthesia and Critical Care, University Hospital Wuerzburg, Oberduerrbacherstrasse 6, 97080 Wuerzburg, Germany; 3 Department of Neurosurgery, University Hospital Magdeburg, Leipziger Strasse 44, 39120 Magdeburg, Germany; 4 Department of Anesthesiology, Klinikum Kassel, Moenchebergstrasse 41-43, 34125 Kassel, Germany; 5 Department of Neuroradiology, University Hospital Wuerzburg, Josef-Schneider-Strasse 11, 97080 Wuerzburg, Germany

**Keywords:** Neurology, Cerebrovascular disorders, Neurovascular disorders, Clinical trials, Phase II trials

## Abstract

Temporary hypercapnia has been shown to increase cerebral blood flow (CBF) and might be used as a therapeutical tool in patients with severe subarachnoid hemorrhage (SAH). It was the aim of this study was to investigate the optimum duration of hypercapnia. This point is assumed to be the time at which buffer systems become active, cause an adaptation to changes of the arterial partial pressure of carbon dioxide (PaCO_2_) and annihilate a possible therapeutic effect. In this prospective interventional study in a neurosurgical ICU the arterial partial pressure of carbon dioxide (PaCO_2_) was increased to a target range of 55 mmHg for 120 min by modification of the respiratory minute volume (RMV) one time a day between day 4 and 14 in 12 mechanically ventilated poor-grade SAH-patients. Arterial blood gases were measured every 15 min. CBF and brain tissue oxygen saturation (StiO_2_) were the primary and secondary end points. Intracranial pressure (ICP) was controlled by an external ventricular drainage. Under continuous hypercapnia (PaCO_2_ of 53.17 ± 5.07), CBF was significantly elevated between 15 and 120 min after the start of hypercapnia. During the course of the trial intervention, cardiac output also increased significantly. To assess the direct effect of hypercapnia on brain perfusion, the increase of CBF was corrected by the parallel increase of cardiac output. The maximum direct CBF enhancing effect of hypercapnia of 32% was noted at 45 min after the start of hypercapnia. Thereafter, the CBF enhancing slowly declined. No relevant adverse effects were observed. CBF and StiO_2_ reproducibly increased by controlled hypercapnia in all patients. After 45 min, the curve of CBF enhancement showed an inflection point when corrected by cardiac output. It is concluded that 45 min might be the optimum duration for a therapeutic use and may provide an optimal balance between the benefits of hypercapnia and risks of a negative rebound effect after return to normal ventilation parameters.

**Trial registration:** The study was approved by the institutional ethics committee (AZ 230/14) and registered at ClinicalTrials.gov (Trial-ID: NCT01799525). Registered 01/01/2015.

## Introduction

Cerebral vasospasm and delayed cerebral ischemia (DCI) are much-feared complications and the main reason for prolonged hospitalization and persistent disabilities after aneurysmal subarachnoid hemorrhage (SAH). Cerebral blood flow (CBF) is—under physiological conditions—regulated by autoregulation as reactivity to changes of transmural wall tension, by equilibrium of vessel relaxing and constricting factors and by changes of the arterial partial pressure of carbon dioxide (PaCO_2_). While autoregulation is deranged and the balance of paracrine factors shift towards vessel contraction, the PaCO_2_ reactivity may be altered but is, in principle, intact after aneurysmal SAH^1,2^. Elevated PaCO_2_ can cause vasodilation of cerebral vessels via the carboanhydrase pathway, which, as a result of increasing pCO_2_, leads to a lowered pH in the CSF, which in turn leads to relaxation of the vascular smooth muscle cells. Another way is through direct interaction of PaCO_2_ with the endothelium of the cerebral vascular system^3^. The degree of reactivity of CBF after SAH has been shown to be an indicator for the course of the disease after SAH with poor reactivity indicating a high risk for delayed cerebral ischemia and poor prognosis. During delayed vasospasm of proximal intracranial vessel trunks, *hyperventilation* induces ischemia and infarction due to an additional constriction of small distal blood vessels and is associated with poor outcome after SAH^4,5^. However, previous trials investigated only minor PaCO_2_ variations around the normal range. Petridis and co-workers have demonstrated that permissive hypercapnia is not hazardous to patients after SAH if they have an external ventricular drainage (EVD) continuously draining cerebrospinal fluid (CSF) during the period of hypercapnia^6^. In a previous trial, it was demonstrated that more pronounced changes of PaCO_2_ can reproducibly affect CBF even after severe SAH suggesting a therapeutic potential of temporary hypercapnia^7^. However, it is well known that alterations of arterial blood gases are followed by adaptation mechanisms. Research data on high altitude adaptation showed that the effect of increased CBF due to hypercapnia is timely limited^8,9^.

The optimum duration of controlled hypercapnia and its effects on CBF and brain tissue oxygen saturation (StiO_2_) as a therapeutic tool against DCI are not clarified. In a previous proof-of-principle study^7^, the CBF reactivity was demonstrated in patients with poor-grade SAH. A stepwise increase of PaCO_2_ in 15 min intervals up to 60 mmHg resulted in a concordant increase of CBF, which did not result in a negative rebound effect, but a sustained increase of CBF after resetting the respiration volume to baseline parameters. The questions addressed in the present work were at what point the beneficial effects of long-term hypercapnia would be exhausted or reverse to possible negative effects. The measurements of CBF and StiO_2_ as the primary and secondary endpoints were performed invasively with an intracerebral thermodilution probe and noninvasively by bifrontal near-infrared spectroscopy, respectively. In this prospective trial we aimed to investigate the course of CBF during longer-term hypercapnia and determine the time-point at which physiologic adaptation mechanisms start to mitigate the CBF-increasing effect in order to locate the optimum duration of controlled hypercapnia in poor-grade SAH patients.

## Material and methods

The study was approved by the institutional ethics committee (AZ 230/14, Ethics committee of the medical faculty, University of Wuerzburg, Germany) and registered at ClinicalTrials.gov (Trial-ID: NCT04687605, registered 01/01/2015). All methods were performed in accordance with the guidelines and regulations of the institutional ethics committee. Also, the study has been conducted in accordance with the ethical standards laid down in the 1964 Declaration of Helsinki and its later amendments. Written consent of a legal guardian was obtained for each patient prior to inclusion into the study.

### Inclusion criteria

Patients were eligible for recruitment if they had suffered severe aneurysmal SAH (Hunt/Hess grade 3–5, Fisher grade 3) no longer than 96 h ago, had received external ventricular drainage (EVD) due to occlusive hydrocephalus and early treatment of the ruptured aneurysm (coiling or clipping) after a 4-vessel angiography of the cerebral vessels. If no other condition like pulmonary complications after aspiration or elevated intracranial pressure prohibited a reduction of analgosedation after occlusion of the aneurysm, an attempt was made to reduce analgosedation. If the patient did not show appropriate reactions, anaesthetics were reapplied and the patient was included in the study.

### Exclusion criteria

Age under 18 years, pregnancy, non-aneurysmal SAH and the presence of a not occluded aneurysm were exclusion criteria. Furthermore, patients suffering from chronic obstructive lung disease (COLD) were excluded because their cerebrovascular reactivity to changes of PaCO_2_ in the target range of this study is unclear. Patients eligible for inclusion who had an ICP over 20 mmHg were not included and were re-evaluated later for a possible start of the study intervention.

### Safety measures and criteria for an interruption of the study intervention

Study interventions were performed in 24-h intervals (24 ± 1 h). Prior to the daily study intervention, cardiovascular parameters, blood gases and ICP were assessed. For documentation of ICP, the EVD was closed for 5–6 monitor lengths until a steady plateau was reached, then opened again for continuous drainage. If resting ICP was over 20 mmHg at the desired timepoint, the study intervention was not conducted and the patient re-assessed 2 h later. If ICP was still elevated over 20 mmHg, the study intervention was not performed on that day. Criteria to interrupt the trial intervention were predefined as an increase of ICP over 25 mmHg for more than two minutes, hypoxemia (PaO2 < 70 mmHg or sO2 < 90 mmHg) and severe arterial acidosis with an arterial pH-value under 7.25.

### Termination of the study procedures

Study interventions were terminated on day 14. Predefined criteria to terminate the study procedures in an individual patient before day 14 were the cessation of therapy due to a poor prognosis of whatever reason according to the treating physicians’ estimation and if a reduction of analgosedation was possible and the study intervention was no longer feasible due to spontaneous breathing.

### Respiratory settings and study intervention

All patients were intubated and mechanically ventilated as defined by the inclusion criteria. If not indicated for ventilatory reasons anyway, the respirator was brought into a volume-controlled ventilation mode for the daily study intervention (Servo-i, Maquet, Rastatt, Germany) maintaining the same respiratory minute volume (RMV). After a first arterial blood gas analysis (RapidLab, Siemens Health Care, Erlangen, Germany), respirator settings were adjusted to reach a PaCO_2_ within the normal range (36–44 mmHg). This was confirmed by another blood gas analysis. The respiratory rate was then reduced to lower the RMV by 40% in every patient. This value was adopted from a previous study assessing the feasibility and risk profile of the study intervention^7^ and served as a guide value for further respirator adjustments to reach the target PaCO_2_ of 55 mmHg. Arterial blood gases were then measured in 5-min intervals and fine adjustment of the respirator settings made until a steady PaCO_2_ between 50 and 55 mmHg was reached. Thereafter, arterial blood gases were measured in 15-min intervals and further respirator adjustments made in order to maintain a stable PaCO_2_ of 55 mmHg for 120 min. All other respiratory settings (tidal volume, inspiratory-expiratory ratio, and inspiratory fraction of oxygen) remained unchanged. Respirator settings were returned to pre-trial settings to obtain normal PaCO_2_ levels after 2 h.

### Target parameters

Parameters measuring CBF and brain tissue oxygenation were the predefined study endpoints.

Direct measurement of CBF (primary endpoint): The course of CBF, was measured by an intracerebral thermodilution probe (Q-Flow 500®) located in the right frontal cortex. The probe was placed 1.5 cm anterior to the external ventricular drainage and was monitored by a Bowman Perfusion Monitor® (Hemedex™, Cambridge, MA, USA). To obtain a continuous measurement and avoid recalibration during the study intervention, the automatic calibration was paused during the time of the study intervention^10^.

Transcranial Doppler sonography (TCD): Prior to the beginning of each study intervention, baseline measurements of TCD were made. At each time-point during the study intervention, TCD measurements were repeated. Measurements were conducted by a well-trained medical technician otherwise not involved in the study procedures with an experience of over 15 years of TCD measurements.

Cerebral tissue oxygen saturation (S_ti_O_2_) (secondary endpoint): S_ti_O_2_ was measured by bilateral near-infrared spectroscopy (NIRS) electrodes (INVOS®, Covidien, Neustadt/Donau, Germany), which were attached on both sides of the forehead. A baseline value was set prior to the intervention to which the following measurements (every 15 min for 2 h) were compared.

### Physiological parameters

All patients were treated on a neurointensive care unit and received the routine continuous monitoring of cardiovascular parameters including electrocardiography, invasive blood pressure monitoring, monitoring of intracranial pressure via an EVD and central venous pressure (CVP). In patients included into the study, a pulse contour-based measurement of cardiac output (CO) was added to the invasive blood pressure monitoring (proAQT, Pulsion Medical Systems SE, Munich, Germany) in order to follow changes of CO during hypercapnia.

### Protocol and statistical analysis

CBF, S_ti_O_2_, ICP, arterial blood gases, pH and hemodynamic parameters were measured at baseline and at 0, 15, 30, 45, 60, 75, 90, 105 and 120 min of the intervention and documented in a paper-based examination protocol. Acquired data was transferred to Excel®-Worksheets (Microsoft®, Redmont, WA, USA). A Kolmogorov–Smirnov test for data rows was used to decide if a normal distribution can be assumed or has to be rejected. Dynamic changes over time were analysed using a one-way ANOVA for repeated measures. In case of statistical significance, time-points were compared to baseline values using a Dunnett’s post hoc test. Statistical significance was defined if p < 0.05. Statistical analysis was performed using GraphPad Prism 4.0 Statistical Software (GraphPad, La Jolla, CA, USA).

### Ethics approval and consent to participate

The study was approved by the institutional ethics committee (AZ 230/14) and registered at ClinicalTrials.gov (Trial-ID: NCT04687605). Written consent of a legal guardian was obtained for each patient prior to inclusion into the study.

## Results

All 12 patients included in this trial suffered from severe aneurysmal SAH. The average and median Hunt/Hess grade was 4 with four patients classified as Hunt/Hess grade 3, four patients as Hunt/Hess grade 4, and four patients as Hunt/Hess grade 5. All patients had thick subarachnoid blood layers and clots according to grade 3 of the Fisher scale, in ten patients additional intracerebral and/or intraventricular blood was detected in the initial CT scan. Cerebral angiogram at admission revealed a total of 15 aneurysms (3 patients with two aneurysms), of which 13 were occluded within 96 h after ictus, 6 by surgical clipping, and 7 by endovascular coiling. The remaining two aneurysms were not considered to be the origin of SAH in patients with more than one aneurysm. Mean age at admission was 48 ± 7.93 years, 9 were female and 3 were male. Patient characteristics, hemorrhage-related features and location and treatment of the ruptured aneurysms are depicted in Table 1. A total of 106 trial interventions were performed from day 4 to day 14 after SAH.

**Table 1:** Personal, hemorrhage-related and aneurysm-related characteristics of 12 study patients.

Patient number	Sex, age	Hunt/Hess	Fisher	Ruptured aneurysm	Occlusion of rupt. aneurysm	Delayed cer. infarction	GOS 6 months
1	f, 30	4	3 + ICH + IVH	ICA left	Coil	left partial MCA	1
2	f, 56	5	3 + IVH	ICA left	Coil	0	3
3	f, 56	5	3 + ICH + IVH	MCA right	Clip	bilateral MCA	1
4	m, 42	3	3 + ICH	MCA left	Clip	0	4
5	f, 53	5	3 + IVH	Acom	Coil	right partial ACA	4
6	f, 55	3	3	Acom	Coil	0	3
7	m, 49	4	3 + ICH + IVH	Acom	Coil	0	5
8	f, 41	3	3 + ICH	Acom	Clip	0	4
9	f, 56	4	3 + ICH + IVH	Acom	Clip	0	5
10	f, 49	4	3 + ICH	MCA right	Clip	0	5
11	m, 48	5	3 + ICH	MCA right	Coil	0	3
12	f, 44	3	3	ICA left	Coil	0	5

Two patients developed minor secondary infarction (one partial left MCA and one partial right ACA territory). One patient with primary large right temporal ICH developed territorial infarctions in both MCA territories in the course of treatment. Two patients died, one from rebleeding from an endovascularly occluded aneurysm outside of study procedures and one due to extended infarction in both MCA territories.

*ICH* intracerebral hemorrhage, *IVH* intraventricular hemorrhage, *ICA* internal carotid artery, *MCA* middle cerebral artery, *Acom* anterior communicating artery, *GOS* Glasgow Outcome Scale.

### Clinical and radiological course

Mean initial Glasgow Coma Score (GCS) was 8 with deterioration to a GCS of 6 prior to intubation. All patients had developed occlusive hydrocephalus and received an external ventricular drainage (EVD). After occlusion of the aneurysm anesthetics were withdrawn and the patients were allowed to wake up. If regain of consciousness was not appropriate and extubation not possible, anesthetics were readministered and the patients were included in the study after written informed consent was given by a legal guardian. 11 of 12 patients showed a significant increase in daily transcranial Doppler sonography (TCD) and/or Perfusion-CT and underwent follow-up angiography.

### Ventilation and blood gas analysis

**Figure 1:**
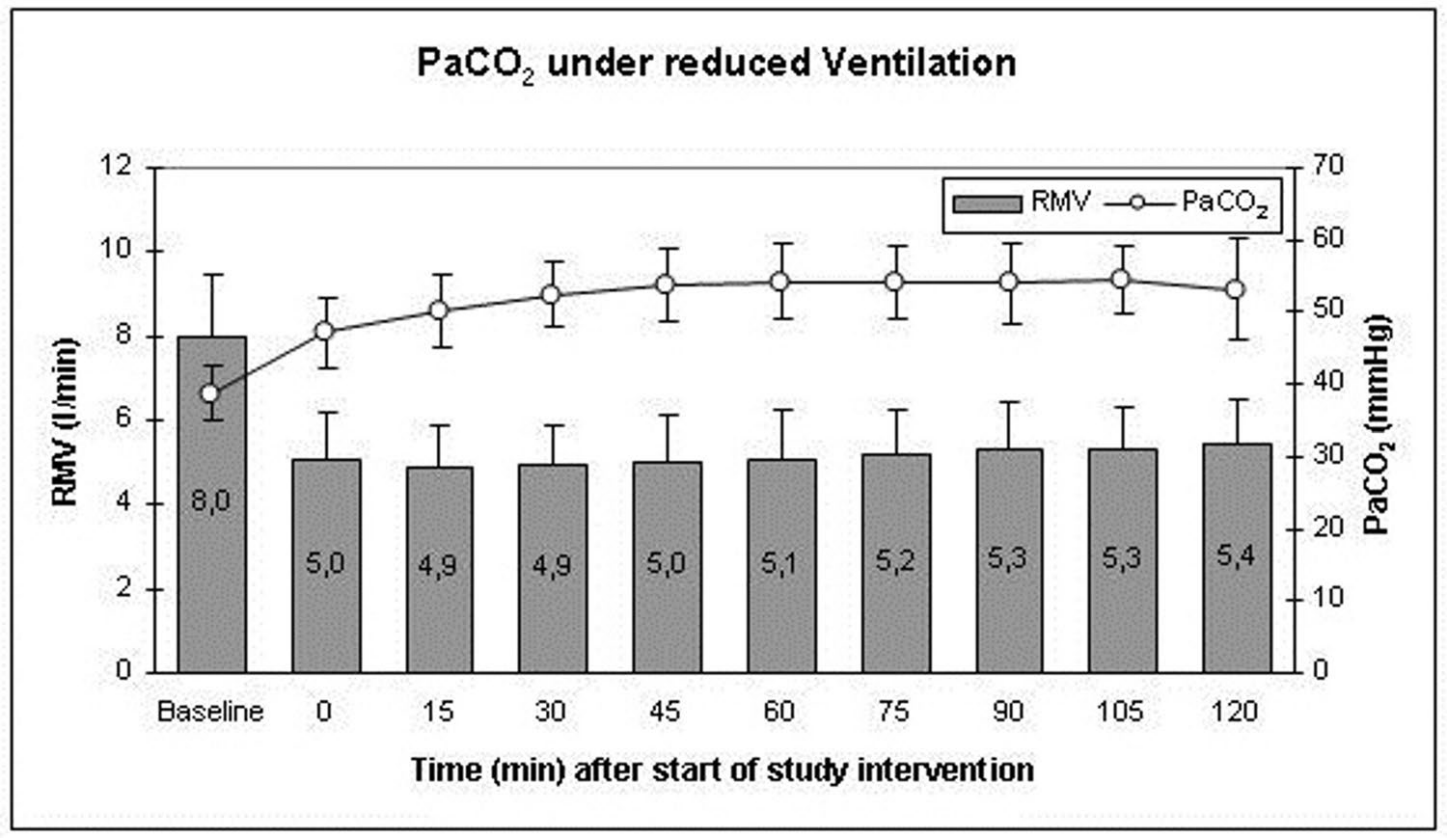
Change of Respiratory Minute Volume (RMV, bars) and concomitant increase of arterial partial pressure of carbon dioxide (PaCO_2_, line plot). At the beginning of the study intervention, RMV was reduced by 40%. Thereafter, minor corrections were made according to blood gas analyses. Data presented as Mean ± SD; 1-way ANOVA for Repeated Measurements; *p < 0.05, **p < 0.01, ***p < 0.001, plots and error bars presented as Mean ± SD (standard deviations).

To reach a target PaCO_2_ of 55 mmHg, the baseline RMV was reduced by 40% in every patient. After the first arterial blood gas control, the respirator settings were further adjusted (Fig. 1). Under this procedure, PaCO_2_ changed from a baseline of 38.75 ± 3.75 mmHg to 47.15 ± 4.81 mmHg at the first measurement under hypercapnia. Thereafter, PaCO_2_ further increased to constantly remain between 50 and 55 mmHg until the end of the trial intervention. Arterial partial pressure of oxygen (PaO_2_) was not influenced by the alterations of the respirator settings with a mean PaO_2_ of 112.55 mmHg (± 24.00 mmHg) before trial intervention and a mean PaO_2_ of 111.67 mmHg (± 13.67 mmHg) after 120 min of hypercapnia. Prolonged hypercapnia led to a decrease in pH as a sign of respiratory acidosis. Nevertheless, no patient reached a pH under 7.25 as a criterion for trial interruption. Mean pH was 7.44 with normocapnia before trial interventions and decreased to 7.28 after 75 min with a slight increase to 7.29 after 90 min, reaching a steady state of 7.29 at 90 to 120 min of hypercapnia (Table 2).

**Table 2:** Arterial blood gas (ABG) values in 15-min intervals after the start of the trial intervention.

	PaCO_2_	pH	PaO_2_
Baseline	38.75 ± 3.65	7.44 ± 0.04	112.6 ± 24.0
Hyp 0 min	47.15 ± 4.81	7.34 ± 0.36	109.8 ± 15.8
Hyp 15 min	50.30 ± 5.02	7.31 ± 0.39	110.7 ± 17.0
Hyp 30 min	52.42 ± 4.13	7.30 ± 0.39	110.3 ± 14.5
Hyp 45 min	53.76 ± 5.17	7.29 ± 0.37	110.6 ± 14.2
Hyp 60 min	54.22 ± 5.27	7.29 ± 0.38	111.9 ± 14.0
Hyp 75 min	54.25 ± 5.06	7.28 ± 0.38	112.2 ± 16.8
Hyp 90 min	54.01 ± 5.51	7.29 ± 0.38	112.3 ± 12.4
Hyp 105 min	54.37 ± 4.66	7.29 ± 0.38	111.4 ± 12.2
Hyp 120 min	53.18 ± 7.16	7.29 ± 0.38	111.7 ± 13.7

After measurement of the baseline value, the respiratory minute volume (RMV) was reduced. The first ABG analysis after reducing the RMV was taken 5 min thereafter to exclude an exaggerated individual effect of the reduction of RMV. “Hyp 0 min” indicates a timepoint 15 min after the start of the study intervention and reduction of RMV. This value was then used to further modify the RMV to reach a target of 55 mmHg. (PaCO_2_ = arterial partial pressure of carbon dioxide, PaO_2_ = arterial partial pressure of oxygen, Hyp + # min indicates the time after the start of the hypercapnic trial intervention).

### Intracranial pressure, arterial blood pressure, cerebral perfusion pressure

**Figure 2:**
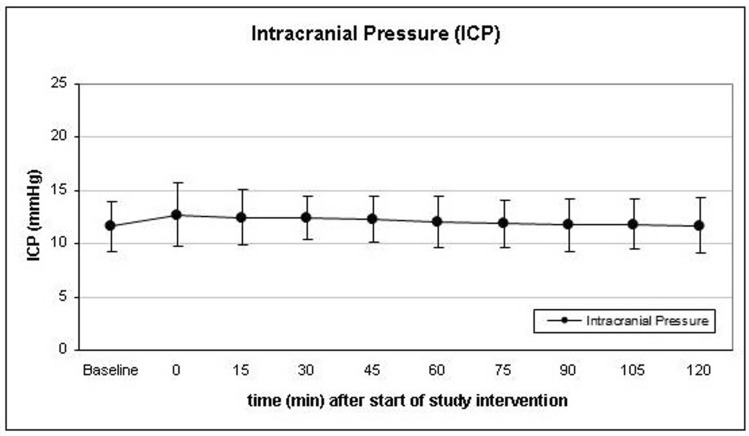
Course of intracranial pressure (ICP) during the study intervention. All patients had an external ventricular drainage which was allowed to continuously drain cerebrospinal fluid during the intervention. No relevant increase of ICP was observed during the monitoring period. Data presented as Mean ± SD; 1-way ANOVA for Repeated Measurements; *p < 0.05, **p < 0.01, ***p < 0.001, plots and error bars presented as Mean ± SD (standard deviations).

Intracranial pressure (ICP) increased slightly but significantly within the first 45 min. Baseline ICP before the start of the study intervention was 11.7 ± 2.4 mmHg at a PaCO_2_ of 38.8 ± 3.7 mmHg. The maximum ICP value of 12.7 ± 3.0 mmHg was recorded immediately after induction of hypercapnia at a PaCO2 of 47.1 ± 4.8. Thereafter, a surplus CSF collection resulted in a continuous decline of ICP thereafter to a final value of 11.8 ± 2.6 mmHg after 120 min (Fig. 2). Mean arterial blood pressure (MABP) increased from a baseline of 100.4 ± 9.6 mmHg to a maximum of 102.2 ± 11.3 mmHg immediately after the induction of hypercapnia and continuously decreased thereafter to reach a final value of 96.0 ± 8.0 mmHg after 120 min. Baseline CPP was 88.6 ± 9.9 mmHg to increase to a maximum of 89.5 ± 11.2 mmHg after induction of hypercapnia and continuously decrease to 84.2 ± 7.9 mmHg at the end of the monitoring time. The decreases of MABP and CPP both were significant from 60 min until 120 min after induction of hypercapnia.

### Cerebral tissue oxygenation

**Figure 3:**
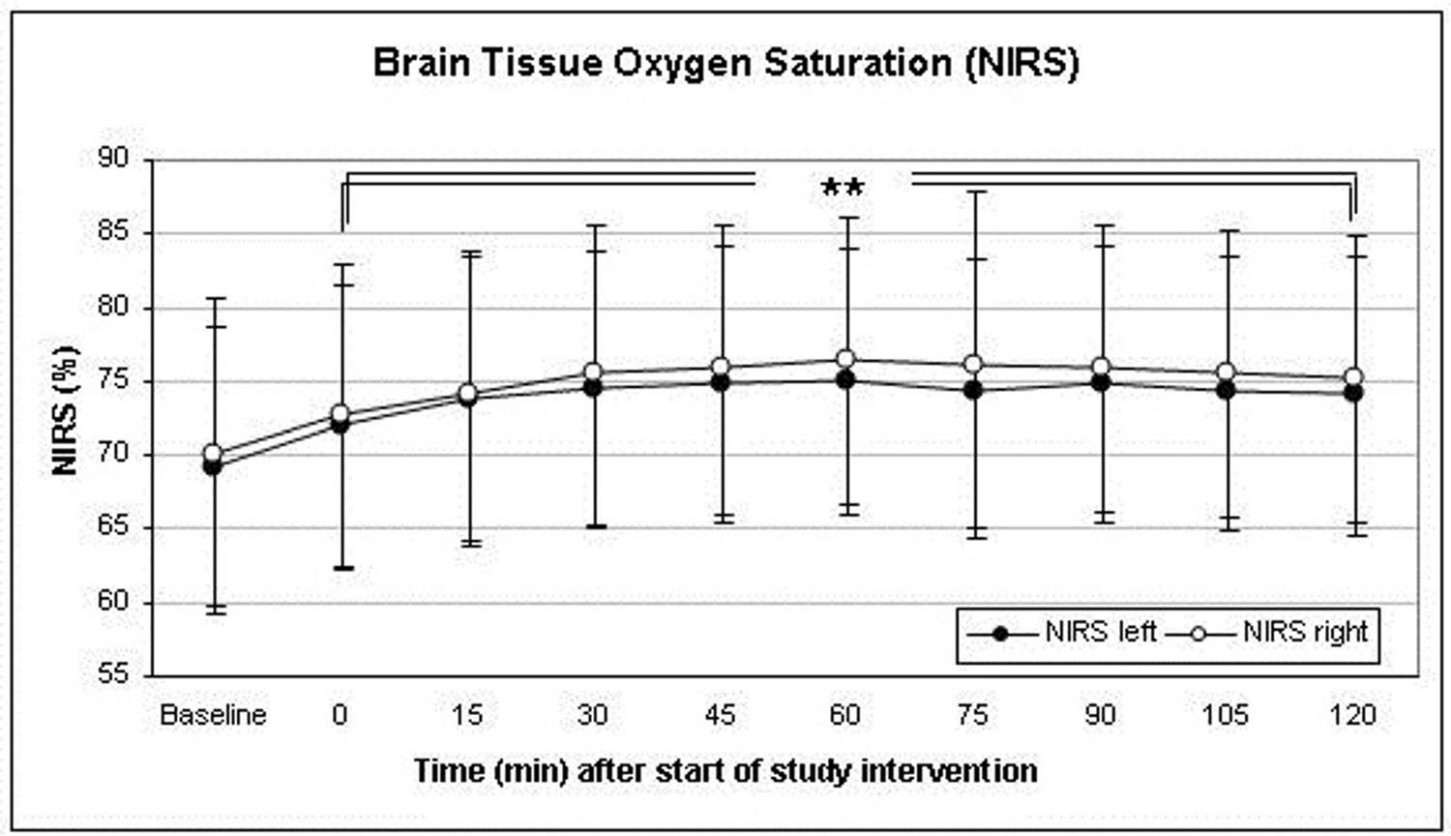
Changes of cerebral tissue oxygen saturation (S_ti_O_2_) was measured by near infrared spectroscopy (NIRS). S_ti_O_2_ increased over both hemispheres with a peak after 60 min. Thereafter, the beneficial effect of the study intervention seemed to weaken. (*p < 0.05, **p < 0.01, ***p < 0.001). Data presented as Mean ± SD; 1-way ANOVA for Repeated Measurements; *p < 0.05, **p < 0.01, ***p < 0.001, plots and error bars presented as Mean ± SD (standard deviations).

S_ti_O_2_ measured over the right forebrain increased to a maximum of 109 ± 8.8% of baseline 60 min after induction of hypercapnia at a PaCO_2_ of 54.22 ± 5.27 mmHg. Over the left forehead, S_ti_O_2_ increased to a maximum 109 ± 7.9% of baseline after 60 min into the trial intervention, respectively. The course of S_ti_O_2_ during hypercapnia is depicted in Fig. 3.

### Transcranial Doppler sonography

**Figure 4:**
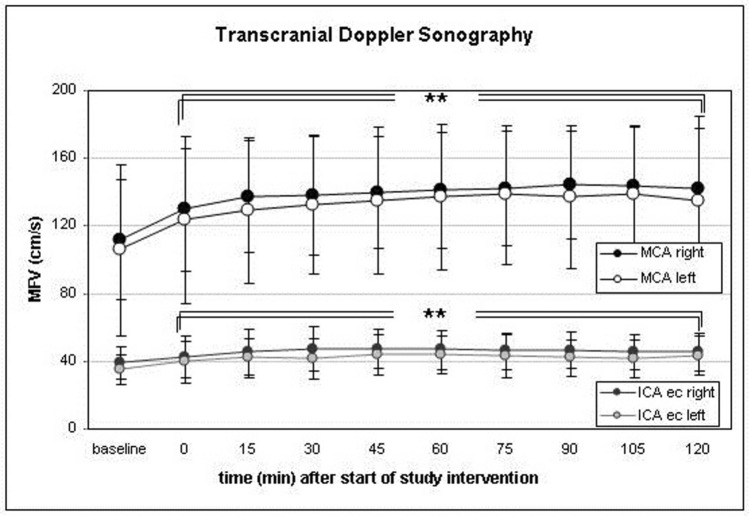
Mean flow velocities (MFV) in transcranial Doppler sonography (TCD) increased during the study intervention in all intracranial vessels. The graph exemplarily depicts the course of MFV in both middle cerebral arteries. The parallel increase of MFV in the high cervical extracranial internal carotid artery (ICA ec) indicates that the increase of flow velocities is due to a global increase of cerebral blood volume and perfusion. (*p < 0.05, **p < 0.01, ***p < 0.001). Data presented as Mean ± SD; 1-way ANOVA for Repeated Measurements; *p < 0.05, **p < 0.01, ***p < 0.001, plots and error bars presented as Mean ± SD (standard deviations).

Mean flow velocities (MFV) in TCD increased in both middle cerebral arteries during the trial intervention. In the right middle cerebral artery (MCA), MFV increased from a baseline of 112 ± 35 cm/s to a steady level of 140–145 cm/s between 45 and 120 min into the trial intervention. In the left MCA, MFV increased from a baseline of 106 ± 51 cm/s to values between 135 and 140 cm/s from 45 to 120 min after induction of hypercapnia. The increase was significant throughout the entire period of monitoring (Fig. 4).

### Cardiac output

During the trial intervention, cardiac output (CO) increased from a baseline of 6.3 ± 2.0 l to 6.6 ± 2.0 l (106%) after induction of hypercapnia, 7.1 ± 2.3 l (114%) after 45 min and 7.5 ± 2.5 l (120%) after 120 min. This increase was caused by an equal 10% increase of stroke volume and heart rate.

### Cerebral blood flow

**Figure 5:**
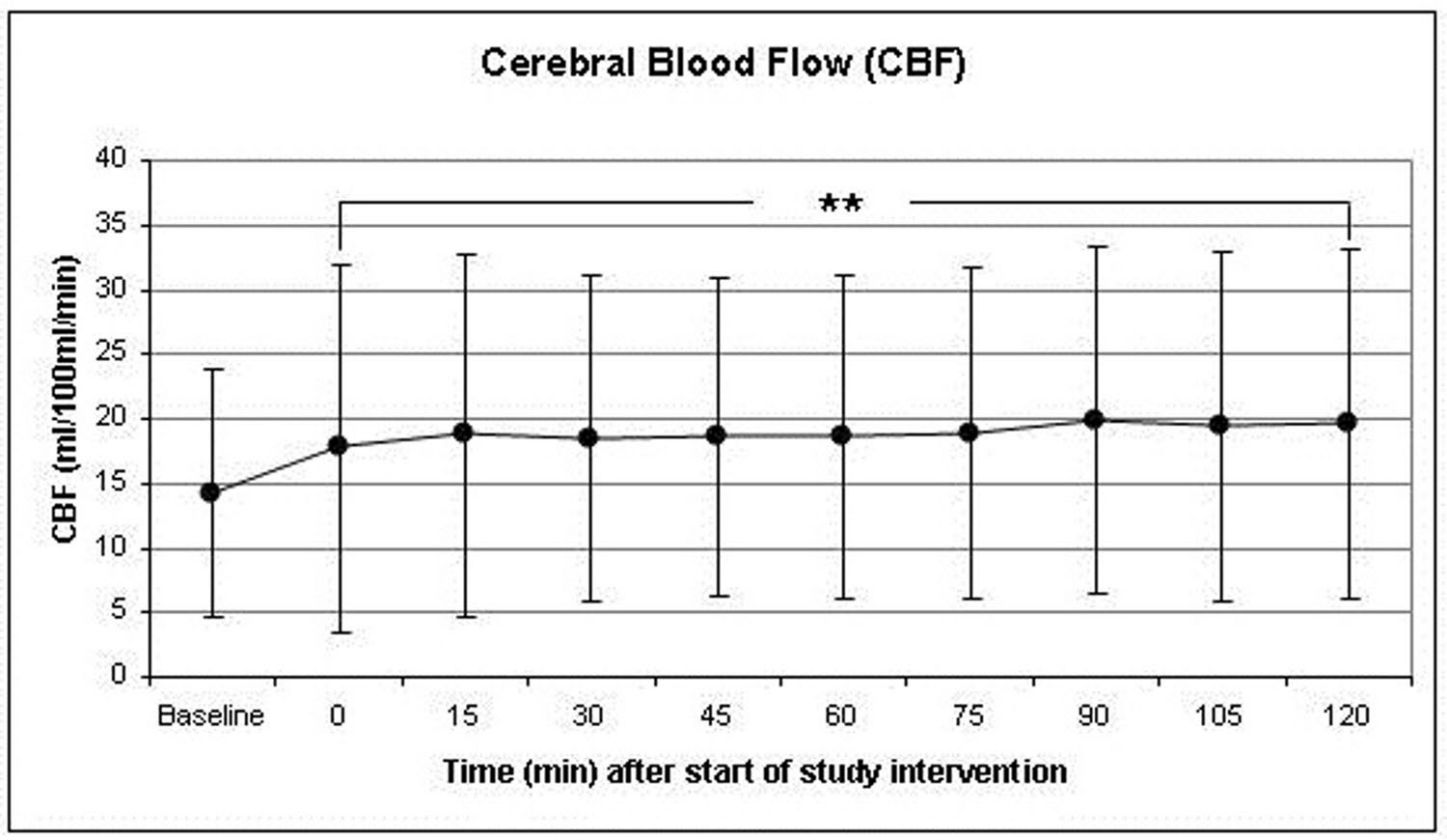
The course of cerebral blood flow (CBF) during the study intervention was measured by a right frontal intracerebral thermodilution probe. (*p < 0.05, **p < 0.01, ***p < 0.001). Data presented as Mean ± SD; 1-way ANOVA for Repeated Measurements; *p < 0.05, **p < 0.01, ***p < 0.001, plots and error bars presented as Mean ± SD (standard deviations).

**Figure 6:**
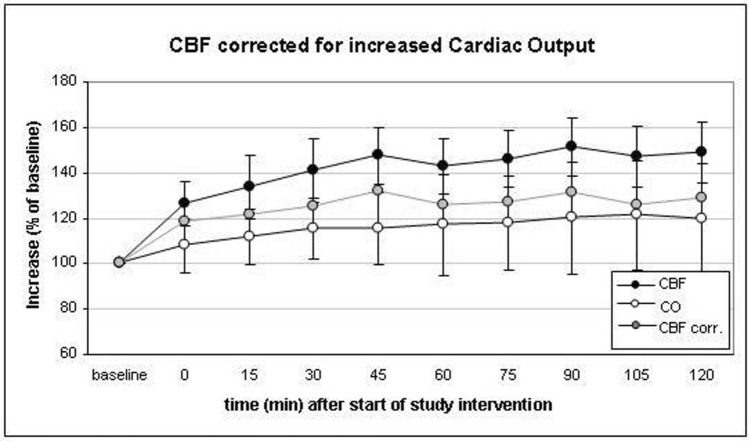
Time course of cerebral blood flow (CBF, black dots), cardiac output (CO, white dots) and CBF corrected for CO (grey dots). The correction subtracts the increase of CO from the increase of CBF in order to calculate the intrinsic CBF-increasing effect of the study intervention in the cerebral vasculature. The peak increase was noted after 45 min. Data presented as Mean ± SD; 1-way ANOVA for Repeated Measurements; *p < 0.05, **p < 0.01, ***p < 0.001, plots and error bars presented as Mean ± SD (standard deviations).

Increasing PaCO_2_ from a baseline value of 38.77 ± 3.75 mmHg to 53.18 ± 7.16 mmHg after 120 min resulted in a final increase of cerebral blood flow (CBF) to149 ± 93% of baseline. After 30, 45, 60, 75 and 90 min CBF increased to 141 ± 92%, 146 ± 99%, 140 ± 80%, 144 ± 80%, 151 ± 86%, and 147 ± 93% of baseline values, respectively. The peak value was recorded after 75 min (Fig. 5). In order to calculate the intervention’s direct effect upon the brain vasculature, the increase of CBF was corrected by the concomitant increase of CO resulting in a maximum net increase of CBF to 132% of baseline after 45 min and decreased thereafter to reach a final value of 129% of baseline at the end of the monitoring period (Fig. 6).

## Discussion

In a previous phase 1 trial, it was observed that CBF can be reproducibly increased by intermittent controlled hypercapnia in the days following aneurysm rupture in patients with poor grade SAH^7,11^. In that trial, CBF increased during a sequential increase of arterial PaCO_2_. After resetting mechanical ventilation to baseline parameters, CBF showed a slow and asymptotic return to starting levels without a negative rebound effect. This observation suggested that a longer duration of hypercapnia might even extend the CBF-increasing effect. The present study was planned as a dose optimization study in order to identify the time-point at which CBF reaches a maximum. It was assumed that after a maximum CBF increase upon elevated PaCO_2_, buffer mechanisms in blood and CSF may result in adaptation mechanisms that lead first to an attenuation of the CBF-increasing effect and then to a negative rebound effect after the hypercapnic challenge is terminated. For safety reasons, a preliminary termination of the study intervention was included into the study protocol if the CBF-enhancing effect secondarily declined by more than 25%. In order to identify the time-point of the maximum intrinsic effect of controlled hypercapnia upon the cerebral vasculature, the value was corrected by the possible inotropic effect of prolonged hypercapnia which has been described previously^12^. Indeed, a positive inotropic effect was also noted during the study interventions conducted in this present study, moderate but statistically significant. It resulted in a “net” optimum effect of the hypercapnic challenge on the cerebral vasculature of 45 min. After the proof of principle in a previous study and the present results we suggest that this may be the basis for an evaluation of efficacy in a controlled clinical trial.

Aneurysmal SAH is still a life-threatening incident with poor over-all prognosis. Its course is characterized by serially occurring ischemic events. Immediately after the rupture of the aneurysm, cerebral perfusion pressure (CPP) decreases due to a sudden increase of ICP resulting in a reduction of CBF and global ischemia. Simultaneously or shortly thereafter, diffuse early arterial vasoconstriction has been observed and contributes to the persistent perfusion deficit in the early stage of SAH^13,14^. Due to developments in emergency care, an increasing number of patients with poor-grade SAH can be adequately resuscitated and find their way to a specialized unit. Patients in a poor clinical condition and large amounts of blood in the subarachnoid space, in turn, are prone to develop vasospasm and delayed cerebral ischemia. To date, no single drug or manipulation has proven undoubtedly effective to prevent or treat the risk for delayed cerebral ischemia (DCI). Even the evidence for nimodipine, the most widely used pharmacological attempt to prevent DCI is based on a single trial which was conducted more than 30 years ago before endovascular aneurysm and vasospasm therapy was invented and without intensive care therapy with contemporary standards^15,16^.

Delayed vasospasm after a few days, caused by endothelial dysfunction, structural changes of the vessel wall, and inflammatory processes is a decisive factor for delayed ischemic neurological deficits (DIND) and delayed cerebral ischemia (DCI)^17,18^. A variety of clinical and experimental studies have investigated various approaches to prevent vasospasm^15,19–21^. But even initially promising trials, e.g. with endothelin A receptor antagonists, successful in treating vasospasm, did not prevent DCI or improve clinical outcome^22^.

Most likely, DCI is a multifactorial phenomenon with delayed cerebral vasospasm, cortical electrical hyperactivity with enhanced energy expenditure, disturbed equilibrium of local vasodilatory and vasoconstrictive paracrine factors and a possible primary metabolic derangement contributing to ischemic damage. However, these factors finally turn into a common finish line, the mismatch of oxygen supply and demand of brain tissue resulting in a breakdown of aerobic metabolism, depletion of energy stores and terminal depolarization of neurons and glial cells^23,24^.

From a pathophysiological point of view, delayed ischemia after SAH is a multifactorially conditioned slowly arising perfusion deficit rather than a sudden and complete ischemia due to severe vasospasm^23,25,26^. Energy depletion, therefore, is also likely to be gradual. Thus, the rationale behind intermittent controlled hypercapnia is to temporarily increase a critically reduced CBF so that energy stores can recover.

It has long been shown that cerebral autoregulation of arterial blood pressure is disturbed after severe aneurysmal SAH and that the equilibrium of local factors are shifted towards constriction. However, the reactivity to changes of PaCO_2_ remains vital^2,4,27,28^. Increased PaCO_2_ may not have an effect on CBF and vessel diameter in the early stage of SAH as recently published by Friedrich and co-workers^29^. For the chronic phase, however, previous work has shown a highly reproducible increase of S_ti_O_2_ and CBF under staged hypercapnia. Since this effect was still reproducible during angiographically proven vasospasm^7^ the concept of maximum peripheral vessel dilation downstream of large-trunk vasospasm may have to be rethought. At the same time, it gives space for a therapeutic intervention. A previous phase 1 study has not only shown the proof of principle without noteworthy negative side effects, but also a surprising reduction of the incidence of DCI and relatively favorable outcome as compared to historical controls and data published in literature^7,11^.

The previous results make it seem worthwhile to evaluate this procedure regarding its therapeutic effect. However, to date no data about the ideal duration of hypercapnia on CBF and cerebral tissue oxygenation exists. From a physiological point of view these positive effects must be temporary since buffer mechanisms in blood and CSF, connected via the carboanhydrase enzyme, trigger an adaptation to long-term changes of CO_2_^30^.To the best of our knowledge, there is no literature about the long-term effects of hypercarbia upon CBF and the course of its adaptation. From high altitude research it is known that mountaineers start to hyperventilate during ascent, which results in a decrease of CBF. After several hours or days, depending on the altitude, acclimatization processes are leading to a normalization of ventilation and physiological parameters^8,9^. However, this is not the timeframe of interest for the particular needs of our patient collective. In contrast to our study protocol the effects in high altitude and its impact on CBF do not occur in a range of seconds or minutes but rather within hours and days during or after an ascent. An additional factor is the lower partial pressure of O_2_ in high altitude leading to hypoxia; therefore, this data is not comparable with normoxic conditions on an intensive care unit. Adaptation mechanisms to long-term hypercapnia, e.g. buffer in the cerebrospinal fluid or blood via carboanhydrase are neither well known nor investigated in trials up to now. Alterations of the pH of the brains’ extracellular space as a regulator of the cerebrovascular response to CO_2_ were discussed by Lassen et al.^31^. Yoon et al. suggest that CBF is regulated by pH-changes of the CSF and that possibly both, PaCO_2_ and CSF-pH “independently and concomitantly regulate cerebrovascular contractility”. According to their findings, an increase of PaCO_2_ (or a decrease in pH) might directly lead to vasodilatation by hyperpolarization of smooth muscle cells via potassium channels. Indirectly, vasodilatation could occur through endothelium-dependent mechanisms^3^. Raichle and co-workers examined the effects of transient *hyperventilation* for several hours on CBF in healthy volunteers covering the period that is of interest for our particular study setting. They showed that CBF decreased with the onset of hyperventilation, but gradually recovered again under continuous hyperventilation and finally exceeded baseline levels in terms of a rebound effect when ventilation was returned to normal. PaCO_2_ also decreased initially but remained constantly low under hyperventilation and returned to baseline parameters after ending of hyperventilation^32^.

We assume that the time-course, extent and reaction upon adaptation to *hypercapnia* is approximately the same as the adaptation to *hyperventilation* as reported by Raichle et al.^32^. This implies that in patients with SAH, whose CBF is critically reduced, there is the danger that a negative rebound effect may carry CBF below ischemic thresholds even if there is a beneficial effect during the study intervention. Therefore, the duration of CBF hypercapnia must not exceed a safe time-frame. The additional measurement of S_ti_O_2_ as a secondary endpoint was therefore mainly because the CBF measurement only covers the right frontal lobe and is therefore more or less a punctual measurement. Bilateral NIRS, on the other hand, covers both MCA and ACA territories, albeit only as a relative indicator for an increase in CBF. NIRS, as well as TCD, however, showed a very good correlation with the right frontal CBF values. Thus, we can conclude that the effect of hypercapnia on CBF is 1. not only selective, and 2. not a steal effect from critically perfused areas into “healthy” territories.

Previous data on the effect of graded hypercapnia after aneurysmal SAH showed a sustained effect on CBF and S_ti_O_2_ and no rebound effects or adverse events^7^. In this present trial, under continuous hypercapnia of 50–55 PaCO_2_ for 120 min, CBF and S_ti_O_2_ reached peak values after 75 min and 60 min, respectively. Hence, at first glance 60 to 75 min seem to be the ideal duration for the highest treatment benefit. Regarding the parallel increase of cardiac output under prolonged hypercapnia the results have to be corrected in order to calculate the maximum intrinsic effect upon cerebral vessels. As a result of disturbed cerebral autoregulation after severe SAH, systematic increase of cardiac output alone, e.g. via application of catecholamines, could lead to an increase in CBF. In the face of disrupted autoregulation, changes of blood pressure and cardiac output are transferred into brain circulation as well as into other parts of systemic circulation^33^. Accordingly, we found that blood flow in the high cervical extracranial carotid artery increased during study intervention (Fig. 4). Therefore, we find it necessary to correct the gross increase of CBF for the increase of CO in order to determine the net impact of the hypercapnic challenge on cerebral perfusion. Adjusting CBF by cardiac output, the hypercapnia-induced net increase of CBF was highest as early as 45 min after the start of the trial intervention. Given the statistically significant elevation of cardiac output after 60 min with further increase under prolonged hypercapnia, sustained therapeutic hypercapnia after reaching its maximum effect on CBF seems not advisable; adaptation mechanisms and negative cardiac effects may bear the danger of a negative rebound effect. The latter was not registered in this present study after 120 min of hypercapnia. However, these observations are based on a few cases and interventions. The reason for this is a practical one. This is not an experimental but an ICU setup and the patients are unexceptionally in a critical condition. Thus, they are not available for study purposes in an unlimited extent. Before and after the 2-h study intervention, a variety of medical and nursing measures are necessary which withdraw the patient from specific monitoring measures. For further TCD-monitoring this is a lack of access to the patient. For the primary endpoint it is rather a technical reason since the Hemedex perfusion probe then exceeds the maximum interval the recalibration can be delayed. This interrupts the continuous measurement and may result in incorrect values. Therefore, our conclusion bases on a physiological deliberation. Since a CO_2_ challenge severely interferes with fundamental physiological regulation mechanism it may potentially cause other unwanted side effects. In this particular kind of intervention safety aspects are of utmost priority. Hence, it is the auxiliary concept that the optimum duration of hypercapnia is the time-span before buffer mechanisms cause a secondary decline of CBF under hypercapnia after a primary increase. Graphically this is the inflection point of the CBF-curve. Since it was the purpose to find the optimum intrinsic effect on brain vessels, the increase of cardiac output has to be subtracted from the gross increase of CBF in the face of deranged autoregulation.

## Conclusion

As a result of this study we conclude that 45 min of controlled hypercapnia might be the most suitable duration regarding the CBF-increasing effect, the patients’ safety and the feasibility in an intensive care setting. Considering that a complete normalization of cardiac and physiologic parameters is achieved several hours after returning to baseline ventilation parameters it might be even more beneficial to repeat the CO_2_-intervention at higher frequency, e.g. 2 or 3 times a day, although this has not been explicitly tested in the current trial. A randomized, controlled trial with 45 min of controlled hypercapnia at 12-h intervals is planned for further evaluation of the therapeutic efficacy of this method in poor-grade aneurysmal SAH.

## Data Availability

The datasets analysed during the current study are available from the corresponding author on reasonable request.

## Abbreviations

CBF: Cerebral blood flow
CO: Cardiac output
CPP: Cerebral perfusion pressure
CSF: Cerebrospinal fluid
CVP: Central venous pressure
DCI: Delayed cerebral ischemia
DIND: Delayed ischemic neurological deficits
EVD: External ventricular drainage
GCS: Glasgow Coma Score
ICA ec: Extracranial internal carotid artery
ICP: Intracranial pressure
MABP: Mean arterial blood pressure
MFV: Mean flow velocities
NIRS: Near infrared spectroscopy
PaCO_2_: Arterial partial pressure of carbon dioxide
RMV: Respiratory minute volume
SAH: Subarachnoid hemorrhage
StiO_2_: Brain tissue oxygen saturation
TCD: Transcranial Doppler sonography
